# 

**Published:** 1883-08

**Authors:** 


### CONTENTS.

### COMMUNICATIONS.

Alimentation Among Savage and Civilized Peoples ........................ 357
Chemisty via Dentistry ........................ 378
Mechanical Dentistry ........................ 381
Dental Pathology ........................ 383
Building Fine Houses on Sand ........................ 385
Operating for the Children ........................ 391
Address ........................ 395

### SELECTIONS.

Vertigo ........................ 401
Ear-Ache in Children ........................ 401
Carbolized Iodoform ........................ 402

### CORRESPONDENCE.

American Dental Society of Europe ........................ 402
Pepsin Preparations ........................ 404

### EDITORIAL.

Retarded Growth ........................ 405
A Singular Case ........................ 406
Dental Law in Michigan ........................ 407
Absorbent Cotton ........................ 408

